# Changes in ethics and transparency reporting in dental journal articles between 2021 and 2025: a full-text cross-journal analysis

**DOI:** 10.3389/froh.2026.1901870

**Published:** 2026-08-12

**Authors:** Hadar Better, Thabet Asbi, Gavriel Chaushu, Keren Better, Doron Haim

**Affiliations:** 1 Maccabi-Dent Medical & Quality Assurance Department, Maccabi-Dent, Tel Aviv, Israel; 2 Research and Innovation Department, Maccabi-Dent, Tel Aviv, Israel; 3 Department of Periodontology, Rambam Health Care Campus, Haifa, Israel; 4 Department of Oral and Maxillofacial Surgery, Rabin Medical Center, Petah Tikva, Israel; 5 Department of Information and Computing Sciences, Utrecht University, Utrecht, Netherlands; 6 Health Management Department, Faculty of Health Sciences, Ariel University, Ariel, Israel

**Keywords:** data availability, dental journals, research ethics, text mining, transparency reporting

## Abstract

**Introduction:**

Explicit reporting of research ethics and transparency practices is increasingly emphasized in biomedical publishing, but whether recent changes have occurred uniformly across reporting domains remains unclear.

**Methods:**

We conducted a full-text, cross-journal text-mining study of 240 dental journal articles published in 2021 and 2025, with 20 articles per journal-year stratum across six established dental journals. Full text was screened for eight predefined markers: ethics approval, informed consent, conflict-of-interest disclosure, funding disclosure, data availability, trial registration, animal ethics, and AI/LLM disclosure. Automated detection was checked against single-reader manual coding of a 36-article sample (96.2% agreement). Prevalence was compared using Fisher exact tests. Reported percentages are corpus-level detection rates across all 120 articles per year, not compliance rates among the studies to which each marker applies.

**Results:**

Data availability showed by far the largest change, increasing from 4.2% to 47.5% (*p* < 0.001) and was the only marker with a statistically robust increase. Ethics approval (53.3% to 69.2%), funding disclosure (58.3% to 73.3%), and animal ethics (1.7% to 8.3%) showed smaller directional increases. Conflict-of-interest disclosure was near-universal in both years (95.8% and 97.5%). Informed consent and trial registration showed little change. AI/LLM disclosure was detected only in 2025 (0% to 4.2%). Mean detected markers per article increased from 2.73 to 3.55, and no 2025 article reported zero markers.

**Discussion:**

These findings indicate uneven changes in dental reporting, with the clearest gains in data availability statements and little change in ethics approval, consent, or trial registration.

## Introduction

Over the past decade, biomedical journals and publishers have introduced a succession of structured reporting requirements (1–3) data availability statements, funding disclosures, conflict-of-interest declarations, trial registration, and, more recently, AI-use disclosures (4–9). These requirements are intended to make published research more transparent, accountable, and reproducible. Whether they have translated into practice, and whether any changes have been uniform across reporting domains, is less clear. For those working within a specialty field, the distinction matters: gains driven by submission templates do not carry the same implications as gains in study-level ethics reporting.

Explicit reporting of research ethics and transparency practices has been shaped over the past decade by broader concerns about reducing waste and increasing value in biomedical research (10, 11) and by the parallel development of reporting guidelines coordinated through the EQUATOR Network (5, 12). Statements regarding ethics approval, informed consent, conflicts of interest, funding, data availability, and trial registration are intended to help readers, reviewers, and editors assess how research was governed, disclosed, and made accountable. These statements do not, by themselves, verify the underlying conduct of a study, but their presence and clarity contribute to the transparency with which published research can be interpreted. Because such statements are commonly placed in methods sections, declarations sections, or end matter rather than in abstracts, full-text analysis provides a more appropriate basis for examining whether they are present in published articles.

Dentistry provides a bounded biomedical field in which to examine these reporting patterns. Dental research spans several research contexts — clinical, paediatric, surgical, imaging, laboratory, animal, and review-based — while remaining sufficiently defined to permit a controlled cross-journal comparison. It should not be assumed to represent all biomedical or medical research fields, but it offers a useful case field for evaluating how ethics and transparency reporting appears across established journals and specialties.

Prior work has begun to map transparency practices in dental research. Raittio, Sofi-Mahmudi, and Uribe (13) conducted a programmatic analysis of over 10,000 open-access articles from PubMed-indexed dental journals through 2021, examining five transparency markers across a large and diverse corpus. That analysis found that conflict-of-interest and funding disclosures were present in the majority of articles while data sharing remained rare, and documented broad directional improvement over time. However, the corpus was restricted to open-access articles available in machine-readable format via the Europe PubMed Central database — approximately 3% of all articles published in PubMed-indexed dental journals — and did not include ethics-specific markers such as ethics approval, informed consent, or animal ethics statements. Trends were described across a broad time span without formally comparing two discrete time points. It therefore remains unclear whether reporting practices changed significantly between any specific pair of years, which domains changed and which did not, and whether observed changes reflect gains in study-level ethics reporting or are concentrated in markers more directly shaped by publisher and journal submission infrastructure.

This study examined full-text reporting of eight ethics and transparency markers — ethics approval, informed consent, conflict-of-interest disclosure, funding disclosure, data availability, trial registration, animal ethics, and AI/LLM disclosure — in articles from six established dental journals published in 2021 and 2025. By operating on full-text PDF rather than open-access XML, the analysis was not restricted to articles available via open-access repositories. Formal hypothesis testing with Benjamini-Hochberg correction for multiple comparisons was applied to identify which reporting domains changed significantly between years and which did not.

## Materials and methods

### Study design

We conducted a full-text, cross-journal text-mining study to examine changes in explicit reporting of selected research ethics and transparency markers in dental journal articles published in 2021 and 2025. The analysis was designed to identify reporting statements within published full-text articles, rather than to independently verify whether the underlying ethics approval, consent process, registration, disclosure, or data-sharing practice occurred. The markers were analyzed as corpus-level indicators of explicit reporting prevalence; they were not treated as requirements assumed to apply uniformly to every article type in the corpus.

### Journal selection and corpus construction

Six established dental journals were selected to provide a bounded, cross-specialty case field within dental research. These six journals were purposively selected to represent major dental specialties and high-visibility outlets, forming a bounded case sample rather than a random or exhaustive sample of dental publishing. The selected journals represented orthodontics, clinical dental research, paediatric dentistry, dental research, endodontics, and oral and maxillofacial surgery. For each journal, two publication years were examined: 2021 and 2025. Both are recent, complete publication years. The interval between them spans several policy changes: wider data-availability requirements (2, 3) and new generative-AI disclosure guidance from 2023 onward (4, 7, 9), after large language model writing tools became widely available in late 2022. Four years is wide enough for such changes to appear in published articles, but narrow enough to limit confounding from journal turnover. We compare the two years as separate snapshots. The analysis is descriptive and does not test whether any policy caused the differences. The final corpus included 20 eligible full-text articles per journal-year stratum, yielding 240 articles in total. Journal characteristics, including publisher, ISSN, specialty, and SCImago ranking, are summarised in Supplementary Table S4.

Within each journal-year stratum, the eligible article pool was defined as all original full-text articles formally assigned by the journal to the relevant citation year, excluding editorials, letters to the editor, correspondence items, commentaries, and other non-article publication types. The first twenty eligible articles in chronological order of formal publication within the citation year were retrieved as PDF files for analysis. Articles that appeared online ahead of print in an earlier calendar year but were formally assigned to the relevant citation volume were included in the corresponding stratum. This sampling approach was chosen for tractability; the resulting corpus should be interpreted as a sample of early-citation-year publications within each journal-year stratum rather than a probabilistic sample of all articles within the volume. The sample size was fixed by design and not by a formal calculation: 120 articles per year provide a balanced cross-journal panel, and the precision achieved for each between-year comparison is conveyed by the reported confidence intervals. *Post-hoc* power was not computed, because it is a direct function of the observed *p*-value and adds no independent information.

Before analysis, the corpus was audited for completeness, duplicate files, publication-type eligibility, and publication-year assignment. Duplicate detection was performed using file-level MD5 hashes. The finalized corpus contained 240 eligible PDF files, with 20 articles in each journal-year stratum and no duplicate file hashes. All analyses were performed on lawfully accessed copies of the articles for non-commercial research purposes. Extracted full text is not redistributed, and only aggregate, article-level coded data are reported.

### Text extraction and preprocessing

Full text was extracted programmatically from PDF files using a Python-based workflow. Extracted text was normalized by removing null characters and collapsing repeated whitespace. PDF text encoding varied across publishers: some publishers embedded text with normal inter-word spacing, whereas others stored text as positioned glyphs without reliable word boundaries, so that adjacent words were concatenated (for example, a declaration of conflicts could appear as a single unspaced string). To recover reporting statements regardless of encoding, each marker pattern was matched against both the normalized text and a whitespace-removed copy of the text, so that declarations were detected whether or not the source PDF preserved word spacing. This extraction handling was applied uniformly across the entire corpus and was based on the PDF text format rather than on any article's marker status. Matches arising only from the whitespace-removed stream were carried into the snippet-level quality-control review, where any spurious substring match was checked against the surrounding text and reclassified as absent. For each article, the workflow recorded article-level metadata, including journal, publication year, filename, page count, word count, and extraction status. No extraction failures or exclusions were required in the final corpus.

### Marker definitions

Eight reporting markers were assessed at the article level: ethics approval, informed consent, conflict-of-interest disclosure, funding disclosure, data availability statement, trial registration, animal ethics statement, and AI/LLM disclosure. Each marker was coded as present or absent according to predefined text-mining definitions based on explicit full-text reporting statements.

Because the corpus included heterogeneous article types, individual markers differed in their likely applicability. Trial registration is mainly relevant to interventional studies, animal ethics statements to animal studies, and informed consent to studies involving human participants. The analysis therefore reports explicit marker detection at the corpus level rather than assessing article-specific compliance.

The ethics approval marker captured statements indicating ethics approval, ethical approval, institutional review board approval, ethics committee approval, or equivalent review. The informed consent marker captured explicit reporting of informed consent, written consent, parental or guardian consent, participant consent, or assent. Conflict-of-interest and funding markers captured explicit disclosure statements or statements indicating absence of conflicts or funding. Data availability captured explicit data availability or data sharing statements, including availability on request, in a repository, included in the article, or not generated. Trial registration captured explicit registration language or registry identifiers. Animal ethics captured statements related to animal care, animal use, institutional animal review, or equivalent oversight. AI/LLM disclosure was defined narrowly as an explicit declaration of generative AI, ChatGPT, large language model, or AI-assisted technology use in manuscript preparation; general discussion of AI as a research topic was not coded as AI/LLM disclosure. Marker definitions were informed by the Declaration of Helsinki (14, 15), the CONSORT statement (16, 17), the ARRIVE 2.0 guidelines (18), and the ICMJE framework for trial registration (19). The text-mining approach builds on prior automated assessments of transparency indicators (20); the present study differs in operating on full-text PDF, applying broader marker definitions, and covering a bounded specialty corpus with formal hypothesis testing.

### Text-mining workflow and quality control

Predefined regular expression patterns were applied to the normalized full text of each article. For each article and marker, the workflow recorded the binary marker code, the first matching pattern, and a surrounding text excerpt for quality-control review. Markers with higher risk of false-positive detection underwent additional snippet-level quality control, including AI/LLM disclosure, conflict-of-interest disclosure, data availability statements, and ethics oversight terms.

Snippet-level quality-control review was subsequently extended to the animal ethics marker. All articles initially flagged as animal-ethics-positive were reviewed; four were reclassified as marker-absent because the detected text occurred in citations of animal studies, in discussions of prior literature, in proposals for future research, or in references to systematic-review methodology tools. None contained a declaration of animal use within the reporting article itself. The refined animal ethics counts are reported in Table 1, with unrefined counts retained in the article-level coded dataset for transparency.

**Table 1:** Year comparison of ethics and transparency reporting markers in dental journal articles published in 2021 and 2025.

Marker	2021 *n*/*N* (%)	2025 *n*/*N* (%)	RD pp (95% CI)	*p*	Adj. p
Ethics approval	64/120 (53.3, 44.4–62.0)	83/120 (69.2, 60.4–76.7)	+15.8 (+4.2 to +28.2)	0.017	0.054
Informed consent	49/120 (40.8, 32.5–49.8)	49/120 (40.8, 32.5–49.8)	+0.0 (−12.3 to +12.3)	1.000	1.000
COI disclosure	115/120 (95.8, 90.6–98.2)	117/120 (97.5, 92.9–99.1)	+1.7 (−3.8 to +6.8)	0.722	0.825
Funding disclosure	70/120 (58.3, 49.4–66.8)	88/120 (73.3, 64.8–80.4)	+15.0 (+3.6 to +27.0)	0.020	0.054
Data availability	5/120 (4.2, 1.8–9.4)	57/120 (47.5, 38.8–56.4)	+43.3 (+34.1 to +53.5)	<0.001	<0.001
Trial registration	22/120 (18.3, 12.4–26.2)	17/120 (14.2, 9.0–21.5)	−4.2 (−13.6 to +5.2)	0.484	0.646
Animal ethics^a^	2/120 (1.7, 0.5–5.9)	10/120 (8.3, 4.6–14.7)	+6.7 (+0.2 to +12.3)	0.034	0.068
AI/LLM disclosure	0/120 (0.0, 0.0–3.1)	5/120 (4.2, 1.8–9.4)	+4.2 (−1.1 to +8.1)	0.060	0.096

Values are *n*/*N* (%, 95% Wilson CI). Risk differences: Newcombe hybrid score 95% CI. Adjusted *p*-values: Benjamini-Hochberg procedure across eight primary markers. Marker presence indicates explicit detection in article full text; it should not be interpreted as independent verification of the underlying practice.

a Counts after snippet-level quality-control review; four articles reclassified as absent. RD, risk difference; pp, percentage points; COI, conflict of interest; CI, confidence interval; Adj. p, Benjamini-Hochberg adjusted *p*-value. Only data availability remained significant after adjustment.

Manual coding check. To check whether automated detection was working and to identify where it failed, a stratified random sample of 36 articles (three per journal-year stratum) was selected using a fixed random seed (random_state=42) and coded manually by full-text reading, blind to the automated output. This was a descriptive reliability check rather than a formal reliability study: manual coding was performed by a single author (HB), so the reported agreement reflects concordance between the automated pipeline and one manual reference, not inter-rater reliability of the reference itself. Each marker was coded as present only when the statement pertained to the reporting article itself rather than to a citation, literature discussion, or future-research proposal. Overall agreement was 96.2% (Cohen kappa 0.92) across the 288 marker-article cells. Several markers had very few positive instances in this small sample, so for those markers high agreement largely reflects true negatives and both percent agreement and kappa are unstable; these values should be read as indicative rather than precise.

### Exploratory analyses

Two exploratory analyses were performed. First, explicit mention of an institutional review board, ethics committee, or equivalent ethics oversight body was assessed as a more specific ethics-oversight marker. A stricter exploratory marker for ethics approval number or approval code was also examined, interpreted cautiously because of higher sensitivity to text-mining misclassification.

Second, article-level reporting completeness was summarized as the number of detected markers per article, used descriptively to assess whether reporting density changed between years. For an *a priori* contrast, the eight markers were grouped into publication-infrastructure markers (data availability, funding disclosure, conflict-of-interest disclosure, and AI/LLM disclosure), which are typically implemented through submission templates and editorial policy, and study-conduct markers (ethics approval, informed consent, trial registration, and animal ethics), which depend on the design and conduct of the individual study. The number of markers per article within each group was compared between years using Mann–Whitney U tests. A sensitivity version excluding animal ethics and AI/LLM disclosure was also calculated.

### Statistical analysis

Marker prevalence was summarized overall, by publication year, and by journal-year stratum. For each binary marker, 2-by-2 contingency tables were constructed and Fisher exact tests were used as the primary inferential test. Absolute percentage-point differences (risk differences) were reported with Newcombe hybrid score 95% confidence intervals. Fisher exact *p*-values were adjusted using the Benjamini-Hochberg false discovery rate procedure across the eight primary markers; both unadjusted and adjusted *p*-values are reported in Table 1. The eight between-year marker comparisons constituted the primary comparison family; all other analyses, including the reporting-completeness, marker-grouping, applicable-denominator, ethics-oversight, journal-level, and publisher-level analyses, were exploratory, and their *p*-values should be interpreted descriptively.

For exploratory reporting-completeness indices, differences between 2021 and 2025 were assessed using Mann–Whitney U tests. All analyses were performed using Python (NumPy, SciPy, pandas, matplotlib). Additional details are provided in the Supplementary Methods.

## Results

### Corpus characteristics

The final corpus included 240 full-text articles, comprising 120 articles published in 2021 and 120 in 2025, balanced across six dental journals with 20 eligible articles in each journal-year stratum. All articles were successfully processed for full-text extraction, and the pre-analysis audit identified no duplicate file hashes, no unresolved publication-type exclusions, and no unresolved publication-year assignment flags. The manual check showed 96.2% overall agreement (Cohen kappa 0.92) across the 288 marker-article cells, with the lowest agreement for funding disclosure. For markers with very few positive instances in the sample, agreement was near-complete but largely reflects true negatives and should not be read as evidence of strong detection for those markers.

### Primary marker prevalence by publication year

**Figure 1:**
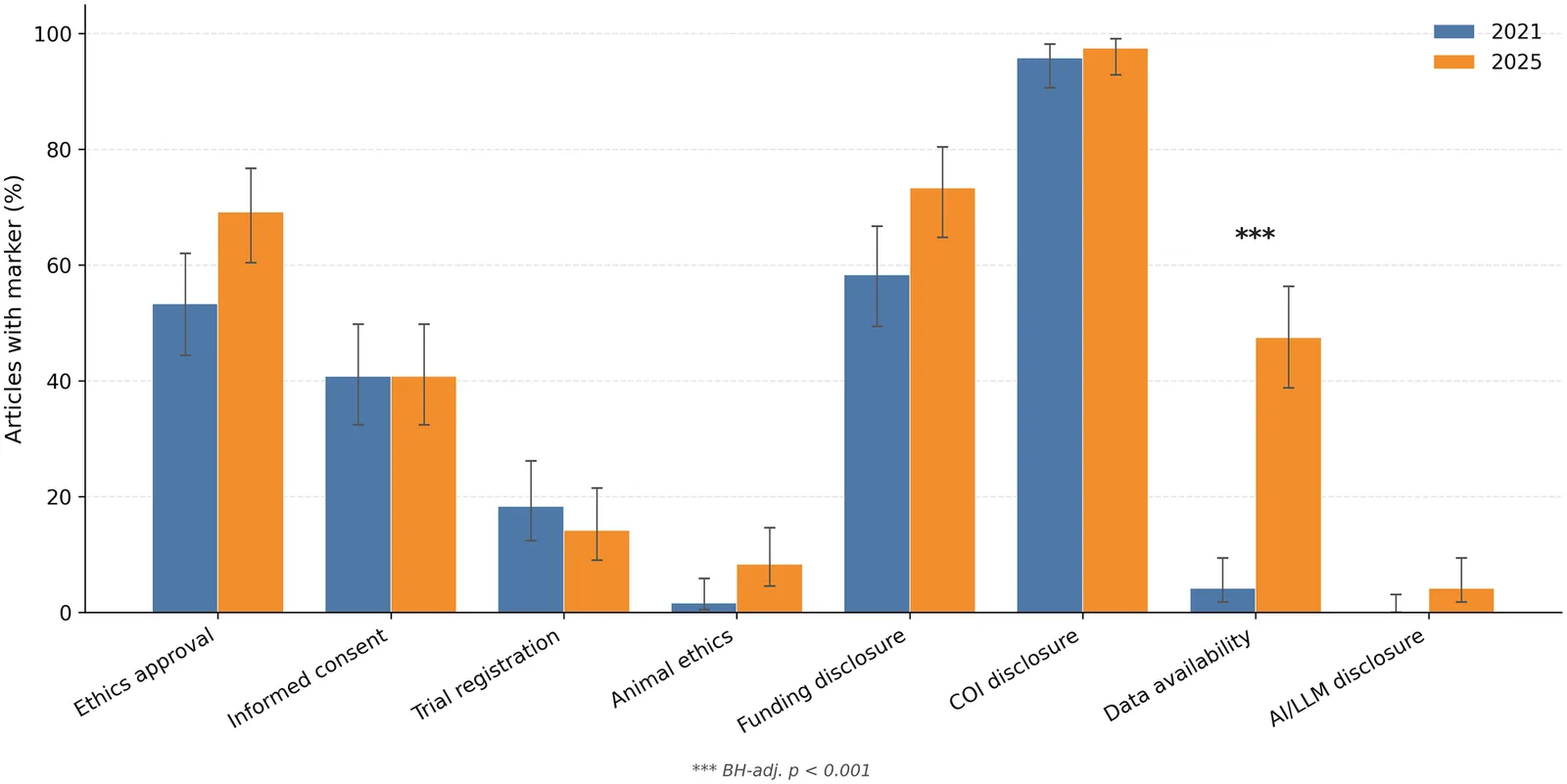
Prevalence of ethics and transparency reporting markers by publication year. Bar chart showing the percentage of articles in which each predefined reporting marker was detected in 2021 and 2025 (*n* = 120 per year). Error bars represent 95% Wilson confidence intervals. The significance marker indicates the Benjamini-Hochberg adjusted *p*-value for the only marker significant after adjustment: *** *p* < 0.001 (data availability).

**Figure 2:**
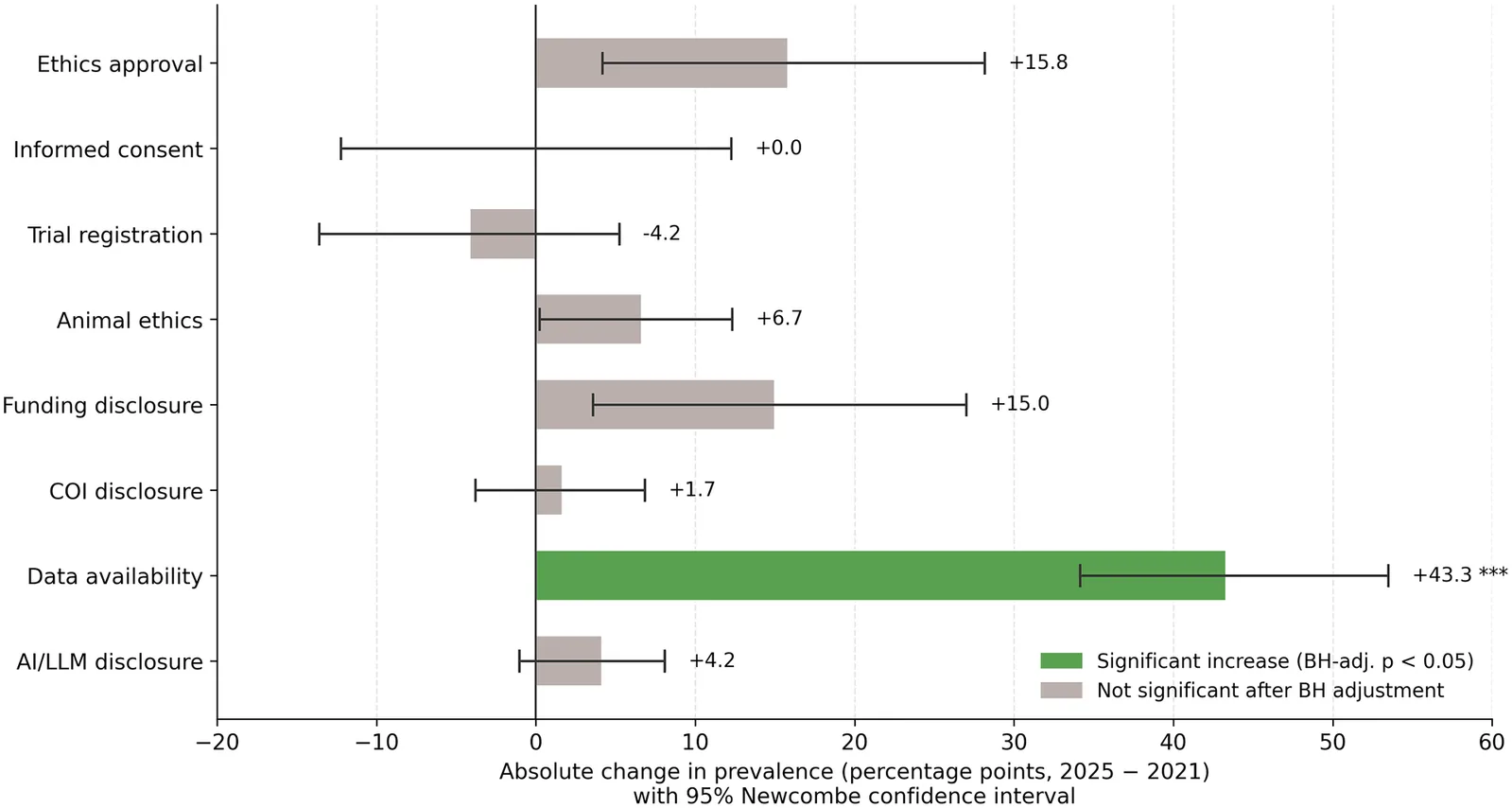
Absolute percentage-point change in reporting marker prevalence from 2021 to 2025. Horizontal bar chart showing the absolute difference in marker prevalence between 2025 and 2021, with 95% Newcombe confidence intervals. Green bars indicate statistically significant increases after Benjamini-Hochberg correction; grey bars indicate non-significant changes.

Reporting markers changed between 2021 and 2025, but the pattern was markedly uneven, with one marker dominating (Table 1; Figures 1, 2). Data availability showed by far the largest temporal change, identified in 5 of 120 articles in 2021 (4.2%; 95% CI 1.8–9.4) and 57 of 120 articles in 2025 (47.5%; 95% CI 38.8–56.4), corresponding to a 43.3 percentage-point increase (95% CI 34.1–53.5; Fisher exact *p* < 0.001; adjusted *p* < 0.001). It was the only marker that remained statistically significant after Benjamini-Hochberg correction.

Ethics approval statements increased from 64 of 120 articles in 2021 (53.3%; 95% CI 44.4–62.0) to 83 of 120 in 2025 (69.2%; 95% CI 60.4–76.7), a 15.8 percentage-point increase (95% CI 4.2–28.2; Fisher exact *p* = 0.017; adjusted *p* = 0.054). Funding disclosure increased from 70 of 120 articles in 2021 (58.3%; 95% CI 49.4–66.8) to 88 of 120 articles in 2025 (73.3%; 95% CI 64.8–80.4), a 15.0 percentage-point increase (95% CI 3.6–27.0; Fisher exact *p* = 0.020; adjusted *p* = 0.054). Both increases were directional but did not retain significance after adjustment.

Conflict-of-interest disclosure was near-universal in both years, appearing in 115 of 120 articles in 2021 (95.8%; 95% CI 90.6–98.2) and 117 of 120 in 2025 (97.5%; 95% CI 92.9–99.1; adjusted *p* = 0.825); the small difference was not significant. Informed consent statements were identified in 49 of 120 articles in 2021 (40.8%) and 49 of 120 in 2025 (40.8%; adjusted *p* = 1.000). Trial registration was identified in 22 of 120 articles in 2021 (18.3%) and 17 of 120 in 2025 (14.2%; adjusted *p* = 0.646).

Animal ethics statements were identified in 2 of 120 articles in 2021 (1.7%) and 10 of 120 in 2025 (8.3%) after quality-control review (Fisher exact *p* = 0.034; adjusted *p* = 0.068); although the relative increase was large, it fell short of significance once adjusted for multiple comparisons. AI/LLM disclosure was identified in 5 of 120 articles in 2025 (4.2%) and none from 2021 (adjusted *p* = 0.096).

### Reporting completeness

Mean detected markers per article increased from 2.73 in 2021 to 3.55 in 2025 (Mann–Whitney U *p* < 0.001; median 3 in 2021 and 4 in 2025; Supplementary Figure S1). Notably, the proportion of articles reporting no markers at all fell from 1.7% in 2021 to 0% in 2025. A sensitivity analysis excluding animal ethics and AI/LLM disclosure showed a similar pattern (mean 2.71–3.42; *p* < 0.001; Supplementary Figure S2).

The grouped contrast reinforced this pattern. Publication-infrastructure markers (data availability, funding, conflict-of-interest, and AI/LLM disclosure) increased from a mean of 1.58–2.23 markers per article between 2021 and 2025 (Mann–Whitney U *p* < 0.001), whereas study-conduct markers (ethics approval, informed consent, trial registration, and animal ethics) did not change significantly (1.14–1.32; *p* = 0.098). The mean gain was more than three times larger for infrastructure markers than for conduct markers (+0.64 vs. +0.18 markers per article). Among marker-article cells that were absent in 2021, infrastructure markers accounted for 77 of the 99 newly reported cells, compared with 22 for conduct markers (Fisher exact *p* < 0.001). This direction held under alternative groupings. Moving funding or conflict-of-interest disclosure to the study-conduct group still left a larger per-article increase for infrastructure markers in every case.

### Journal-level variation

Journal-year analyses demonstrated substantial heterogeneity. In 2025, data availability was identified in 20 of 20 Clinical Oral Investigations articles (100%, 95% CI 84-100), 18 of 20 International Journal of Paediatric Dentistry articles (90%, 95% CI 70-97), and 11 of 20 Journal of Dental Research articles (55%, 95% CI 34-74). These journal-level figures are based on 20 articles per stratum and therefore carry wide confidence intervals; they are descriptive and should not be read as stable estimates. Lower frequencies were observed in AJODO (10%), Journal of Endodontics (15%), and Journal of Oral and Maxillofacial Surgery (15%). This pattern is consistent with infrastructure-driven reporting change operating at the publisher or journal level, rather than with a specific policy: we did not establish the exact timing of any policy change for the included journals, and, because each publisher other than Elsevier is represented by a single journal, journal-specific editorial practices and publisher-level policies cannot be disentangled. Data availability by publisher group is shown in Supplementary Figure S3.

### Exploratory ethics oversight analysis

Explicit mention of an institutional review board, ethics committee, or equivalent ethics oversight body increased from 46 of 120 articles in 2021 (38.3%) to 63 of 120 in 2025 (52.5%; Fisher exact *p* = 0.038). A stricter ethics approval number or code marker increased from 11 of 120 (9.2%) to 24 of 120 (20.0%; *p* = 0.027). These exploratory markers were not specified *a priori* and were not included in the multiple-comparison adjustment; the unadjusted *p*-values are reported for descriptive purposes only. Article-level coding and journal-year summaries are provided in Supplementary Tables S1, S2 and Supplementary Figures S1–S3.

## Discussion

In this full-text cross-journal analysis of selected dental journal articles, explicit reporting of ethics and transparency markers changed between 2021 and 2025, but the changes were strikingly uneven across reporting domains. After adjustment for multiple comparisons, only one marker showed a statistically robust increase: data availability, which rose more than ten-fold. Ethics approval, funding disclosure, and animal ethics showed directional increases that did not retain significance after Benjamini-Hochberg correction. Conflict-of-interest disclosure was near-universal in both years and did not change. Informed consent and trial registration showed little or no change. An exploratory reporting-completeness analysis was consistent with this pattern: the mean number of detected markers per article increased between years, and no 2025 article reported zero markers, compared with 1.7% of 2021 articles. The grouped analysis made this separation explicit: markers tied to submission infrastructure increased significantly per article, whereas markers tied to study conduct did not.

The observed changes are in explicit reporting, not in the underlying ethical conduct of studies. Some markers are closely linked to journal and publisher workflows, whereas others depend on study design, regulatory requirements, participant involvement, or the conduct of the research itself. Recent changes in scholarly publishing appear to have strengthened the visibility of publication-level transparency statements more than they have changed all ethics-related reporting domains in parallel (4, 6).

The largest temporal change was in data availability reporting. Unlike study-specific ethical practices, data availability statements are often implemented through editorial policies, article templates, and submission workflows. The marked increase from 2021 to 2025 may reflect, at least in part, changes in publication infrastructure rather than changes in the underlying data-sharing behavior alone (2, 3, 21, 22). The present analysis identified the presence of data availability statements; it did not assess whether the underlying data were actually accessible, complete, reusable, or consistent with the statement (23, 24). Specialty-specific data sharing infrastructure exists in the dental and craniofacial research community, including FAIR-aligned repositories such as the FaceBase consortium (25). The increase identified here is consistent with changes in reporting infrastructure rather than confirmed deposition in such repositories.

A similar asymmetry has been documented at larger scale. Raittio et al. (13), in a programmatic analysis of over 10,000 open-access articles from PubMed-indexed dental journals through 2021, found that conflict-of-interest and funding disclosures were present in the majority of articles while data sharing remained rare at 2.0%. The present study extends that work in three respects: it operates on full-text PDF rather than open-access XML; it includes ethics-specific markers not previously assessed in dental research; and it applies formal hypothesis testing to a discrete two-year comparison. Where Raittio et al. reported 2.0% data sharing through 2021 using a definition restricted to active data deposition, the present study identified data availability statements in 4.2% of 2021 articles and 47.5% of 2025 articles under a broader marker definition. The difference in magnitude between years is consistent with changes in publication infrastructure during that interval, although the timing and effect of any specific publisher or journal policy were not assessed. Dental-specific studies of CONSORT compliance have likewise documented incomplete reporting (26), suggesting that the patterns observed here are consistent with broader reporting gaps in the specialty. A related Frontiers in Oral Health analysis by Sofi-Mahmudi and Raittio applied a similar automated approach to COVID-19-related dental articles and reported conflict-of-interest disclosure in about three-quarters and funding disclosure in about four-tenths of articles, with data sharing near zero (27); the present study extends that line of work to a broader marker set, a two-year comparison, and manual validation.

Funding disclosure also rose in directional terms, similarly reflecting increasing standardization of end-matter declarations, though this change too was no longer significant once adjusted. Conflict-of-interest disclosure, by contrast, was near-universal in both years (95.8% and 97.5%), consistent with well-established and broadly enforced declaration requirements across the sampled journals. The absence of change therefore reflects a ceiling effect rather than stagnation, and the overall stability should not be interpreted as evidence that author conflict-of-interest practices themselves were unchanged. Conversely, generative-AI disclosure began at zero in 2021 and could only increase; its 2025 rate should therefore be read against that floor, as an emerging practice rather than as a level of compliance.

The journal-level findings reinforce the interpretation that reporting practices are shaped by publication infrastructure. Data availability was identified in all 20 Clinical Oral Investigations articles (Springer Nature) and 18 of 20 International Journal of Paediatric Dentistry articles (Wiley) in 2025, compared with substantially lower rates across the three Elsevier-published journals. Whether the observed clustering reflects publisher policy, journal-level editorial practice, or both cannot be determined from this corpus alone, since each publisher other than Elsevier is represented by a single journal. The publisher and journal composition of the sample is summarised in Supplementary Table S4. The pattern is nonetheless consistent with infrastructure-driven change rather than autonomous improvement in author behavior. This asymmetry suggests that template-based publication requirements alone are unlikely to close the gap between publication-infrastructure markers and study-conduct markers, a separation that was directly supported by the grouped analysis, in which infrastructure markers increased significantly between years while study-conduct markers did not.

Ethics approval statements showed a directional increase between years, with a parallel increase from 38.3% to 52.5% in explicit mention of an IRB or ethics committee (exploratory, unadjusted *p* = 0.038); the primary ethics approval marker did not retain significance after adjustment, and the IRB marker was exploratory and not specified *a priori*. Animal ethics statements also showed a directional increase after quality-control review (from 1.7% to 8.3%; adjusted *p* = 0.068), although absolute numbers were small and the confidence interval was wide. Participant-level consent reporting, by contrast, remained unchanged, suggesting that the broad strengthening of publication-level transparency statements did not extend in parallel to participant-level reporting.

Trial registration remained uncommon and did not increase between years. The corpus included heterogeneous article types, many of which would not be expected to require trial registration, so absence of an increase should not be interpreted as non-compliance. Future studies should evaluate trial registration within a restricted subset of interventional or randomized studies.

Explicit AI/LLM disclosure was rare — identified in only five articles from 2025 and none from 2021. This finding should not be overinterpreted, because formal generative AI disclosure policies are recent and may not have been uniformly implemented during the publication window. It nonetheless suggests that explicit AI-related manuscript-preparation disclosure was not yet common in the sampled dental literature, despite the emergence of living guidelines for the responsible use of generative AI in research (7–9, 28).

The exploratory reporting-completeness analysis provides a useful summary of the overall pattern. Articles published in 2025 contained more detected reporting markers per article than those published in 2021, a pattern that remained in the sensitivity analysis. However, this index should be interpreted descriptively: it is not a validated quality score, and the presence of more reporting statements does not necessarily imply better ethical conduct, better study design, or greater substantive transparency.

This study has several strengths. It used full-text article analysis rather than abstract-only screening, and applied an extraction approach robust to publisher-specific PDF encoding, including documents that store text without reliable word spacing. The corpus was balanced across journal-year strata and audited for completeness, duplicate file hashes, publication type, and formal publication year. Marker definitions were applied consistently and uniformly across the corpus, markers with higher risk of false-positive detection underwent additional quality-control refinement, and automated detection was checked against blinded single-reader manual coding of a stratified random sample. The grouping of markers into publication-infrastructure and study-conduct domains allowed the central question of uneven change to be tested directly rather than described qualitatively.

The study has several limitations. First, the corpus was limited to selected dental journals and should not be interpreted as representative of all dental, medical, or biomedical research. Second, the analysis assessed explicit reporting markers and did not independently verify the underlying practices; absence of a detected marker should be interpreted as absence of explicit reporting, not as evidence that the underlying practice did not occur. Third, some markers were not applicable to all article types, and low prevalence should be interpreted as corpus-level reporting prevalence in a heterogeneous sample. To quantify this, we classified each article by study type and recomputed the design-dependent markers within their applicable subsets (Supplementary Table S3). Corpus-level percentages should therefore not be read as compliance rates. Fourth, the manual validation was a single-reader descriptive check intended to confirm that automated detection was functioning and to locate its weaker points, not a formal inter-rater reliability study. It was not independently double-coded, the validation subset comprised only 36 of 240 articles with few positive instances for several markers, and agreement was lowest for funding disclosure. Percent agreement and kappa for the rarer markers are therefore unstable and should be interpreted with caution; independent double coding of a larger, positive-enriched sample would be needed to quantify detection reliability formally. Fifth, the study compared two publication years rather than continuous time trends, and therefore cannot determine the precise timing or causal drivers of the observed changes. Sixth, the publisher-level analysis is limited by the fact that each publisher other than Elsevier is represented by a single journal, so journal-specific editorial practices and publisher-level policies cannot be disentangled. Seventh, the corpus was restricted to six purposively selected dental journals spanning major specialties; findings may not generalise to the full range of dental publishing. Finally, the study was not pre-registered. The analysis was restricted to extractable text in the main article PDFs; separate supplementary files were not systematically searched, and statements present only in non-extractable images, tables, or Supplementary Material may have been missed. Matches identified only in the whitespace-removed text stream were adjudicated at quality-control review, but their number and the false-positive rate attributable specifically to this step were not recorded separately. We do not report risk ratios, because several markers had very low or very high baseline prevalence, where ratios are unstable. We report absolute risk differences with confidence intervals instead. Eight markers were each tested for between-year change, so *p*-values were adjusted with the Benjamini-Hochberg procedure. Only data availability stayed significant after adjustment. The unadjusted *p*-values in Table 1 are descriptive, not confirmatory.

These findings suggest that template-based submission requirements may improve the visibility of reporting statements. For markers tied to study conduct, journals might consider requiring authors to address ethics oversight explicitly at submission, with the option to state why approval was not applicable for a given study type. Such targeted requirements could help bridge the current gap between easily implemented template-based changes and more substantive improvements in research ethics reporting. Future studies should examine whether the observed increase in data availability statements translates into actual data sharing and reuse, and whether strengthened journal policies can improve reporting of study-conduct markers such as informed consent and trial registration. Overall, this full-text analysis suggests that explicit ethics and transparency reporting in selected dental journals changed between 2021 and 2025, with the change concentrated overwhelmingly in a single publication-level transparency marker, data availability. Other markers, including ethics approval, funding disclosure, and animal ethics, showed directional increases that did not retain statistical significance after Benjamini-Hochberg correction, while participant-level and study-conduct markers showed limited or no change. These findings indicate a markedly uneven evolution in reporting practices and point to a role for journal and publisher infrastructure in shaping the visibility of ethics and transparency information in published research.

## Data Availability

The dataset supporting the conclusions of this article, including the article-level coded data and the analysis outputs, is openly available in Zenodo at https://doi.org/10.5281/zenodo.21397136 under a CC BY 4.0 licence. All other contributions presented in the study are included in the article/Supplementary Material.

## Supplementary material

Supplementary Figure S1 Distribution of reporting completeness, 2021 vs. 2025. Bar chart showing the percentage of articles with each number of detected reporting markers (0–8 scale) in 2021 and 2025. Dashed vertical lines indicate annual means.

Supplementary Figure S2 Sensitivity analysis: distribution of reporting completeness. Bar chart showing the percentage of articles with each number of detected reporting markers on a sensitivity index (0–6 scale) excluding animal ethics and AI/LLM disclosure. Dashed vertical lines indicate annual means.

Supplementary Figure S3 Data availability statement reporting by publisher, 2021 vs. 2025. Percentage of articles with a detected data availability statement in each publisher group. Springer Nature: Clinical Oral Investigations (*n* = 20 per year); Wiley: International Journal of Paediatric Dentistry (*n* = 20 per year); Sage: Journal of Dental Research (*n* = 20 per year); Elsevier: three journals combined (*n* = 60 per year). Significance markers indicate Fisher exact *p*-values: *** *p* < 0.001; ** *p* < 0.01; * *p* < 0.05.
