# Supplementary material for: Changes in ethics and transparency reporting in dental journal articles between 2021 and 2025: a full-text cross-journal analysis
Source: Front Oral Health. 2026 Aug 12;7:1901870. doi: 10.3389/froh.2026.1901870 (PMC13506944; doi:10.3389/froh.2026.1901870)
Supplement: Supplementary file 1 [file Datasheet1.docx]

**Supplementary Table S1. Prevalence of each reporting marker by publication year (validated coding).**

| **Marker** | **2021 (n=120) n (%)** | **2025 (n=120) n (%)** | **2021 95% CI** | **2025 95% CI** |
| --- | --- | --- | --- | --- |
| Ethics approval | 64 (53.3) | 83 (69.2) | 44.4–62.0 | 60.4–76.7 |
| Informed consent | 49 (40.8) | 49 (40.8) | 32.5–49.8 | 32.5–49.8 |
| COI disclosure | 115 (95.8) | 117 (97.5) | 90.6–98.2 | 92.9–99.1 |
| Funding disclosure | 70 (58.3) | 88 (73.3) | 49.4–66.8 | 64.8–80.4 |
| Data availability | 5 (4.2) | 57 (47.5) | 1.8–9.4 | 38.8–56.4 |
| Trial registration | 22 (18.3) | 17 (14.2) | 12.4–26.2 | 9.0–21.5 |
| Animal ethics | 2 (1.7) | 10 (8.3) | 0.5–5.9 | 4.6–14.7 |
| AI/LLM disclosure | 0 (0.0) | 5 (4.2) | 0.0–3.1 | 1.8–9.4 |

*n, number of articles with the marker detected; CI, Wilson 95% confidence interval. Percentages are within-year (denominator 120 per year).*

**Supplementary Table S2. Number of articles with each reporting marker by journal and publication year (validated coding; n=20 per journal-year).**

| **Journal** | **Year** | **Ethics** | **Consent** | **COI** | **Funding** | **Data** | **Trial** | **Animal** | **AI/LLM** |
| --- | --- | --- | --- | --- | --- | --- | --- | --- | --- |
| AJODO | 2021 | 13 | 9 | 18 | 9 | 0 | 2 | 0 | 0 |
| AJODO | 2025 | 14 | 6 | 20 | 9 | 2 | 0 | 1 | 3 |
| Clin Oral Investig | 2021 | 20 | 19 | 20 | 14 | 0 | 4 | 1 | 0 |
| Clin Oral Investig | 2025 | 19 | 15 | 20 | 20 | 20 | 8 | 0 | 0 |
| IJPD | 2021 | 12 | 12 | 20 | 15 | 2 | 7 | 0 | 0 |
| IJPD | 2025 | 15 | 10 | 20 | 20 | 18 | 3 | 0 | 1 |
| JDR | 2021 | 4 | 3 | 20 | 20 | 3 | 4 | 1 | 0 |
| JDR | 2025 | 7 | 3 | 20 | 20 | 11 | 4 | 8 | 0 |
| JOE | 2021 | 4 | 1 | 17 | 6 | 0 | 1 | 0 | 0 |
| JOE | 2025 | 13 | 10 | 17 | 11 | 3 | 2 | 1 | 0 |
| JOMS | 2021 | 11 | 5 | 20 | 6 | 0 | 4 | 0 | 0 |
| JOMS | 2025 | 15 | 5 | 20 | 8 | 3 | 0 | 0 | 1 |

*Each cell is the number of articles (of 20) in that journal-year with the marker detected in full text. Animal ethics counts are after quality-control review. Ethics, ethics approval; Consent, informed consent; COI, conflict-of-interest disclosure; Funding, funding disclosure; Data, data availability; Trial, trial registration; Animal, animal ethics; AI/LLM, AI/LLM disclosure.*
