# Supplementary material for: Changes in ethics and transparency reporting in dental journal articles between 2021 and 2025: a full-text cross-journal analysis
Source: Front Oral Health. 2026 Aug 12;7:1901870. doi: 10.3389/froh.2026.1901870 (PMC13506944; doi:10.3389/froh.2026.1901870)
Supplement: Supplementary file 2 [file Datasheet2.docx]

**Supplementary Material**

**Supplementary Methods**

**Corpus audit and eligibility checks**

The final analysis corpus consisted of 240 full-text articles from six dental journals and two publication years, with 20 eligible articles per journal-year stratum. Before analysis, the corpus was audited to confirm that each stratum contained 20 PDF files, that publication-year assignment corresponded to the formal journal citation year, and that non-article publication types were excluded. Editorials, letters to the editor, correspondence items, commentaries, and other non-article publication types were not included. Duplicate detection was performed using file-level MD5 hashes. Publication-year checks were based on the formal citation year identified in the article, rather than submission, acceptance, copyright, online-first, or epub-ahead-of-print dates.

**Text extraction**

Full text was extracted programmatically from PDF files using a Python-based workflow. Extracted text was normalized by removing null characters and collapsing repeated whitespace. PDF text encoding varied across publishers: some embedded text with normal inter-word spacing, whereas others stored text as positioned glyphs without reliable word boundaries, so that adjacent words were concatenated. To recover reporting statements regardless of encoding, each marker pattern was matched against both the normalized text and a whitespace-removed copy of the text. This extraction handling was applied uniformly across the entire corpus and was based on the PDF text format, not on any article's marker status. Full-text PDF files and extracted full-text article content are not redistributed in the supplementary material.

**Marker detection**

The analysis assessed eight article-level reporting markers: ethics approval, informed consent, conflict-of-interest disclosure, funding disclosure, data availability statement, trial registration, animal ethics statement, and AI/LLM disclosure. Each marker was coded as present or absent based on explicit full-text reporting statements. Absence of a detected marker should be interpreted as absence of explicit reporting according to the predefined text-mining criteria, not as evidence that the underlying practice did not occur.

**Quality-control refinement**

For each article and marker, the workflow generated a binary marker code and a surrounding text excerpt for internal quality-control review. Markers at higher risk of false-positive detection underwent additional refinement. The AI/LLM marker was refined to capture explicit disclosure of generative-AI or AI-assisted writing-related use rather than articles that studied artificial intelligence as a research topic. For the animal ethics marker, all articles initially flagged as positive were reviewed; three were reclassified as marker-absent because the detected text occurred in citations, literature discussion, or future-research proposals rather than in a declaration of animal use within the reporting article itself.

**Validation against manual coding**

To assess the reliability of automated detection, a stratified random sample of 36 articles (three per journal-year stratum) was selected using a fixed random seed (random_state = 42) for reproducibility and independently coded by manual full-text reading, blind to the automated output. Each of the eight markers was coded as present or absent using the same definitions as the automated workflow, with a marker counted as present only when the statement pertained to the reporting article itself rather than to a citation, literature discussion, or future-research proposal. Agreement between manual and automated coding was 96.2% overall (Cohen kappa 0.92) across the 288 marker-article cells, with per-marker agreement ranging from 88.9% (funding disclosure) to 100% (conflict-of-interest disclosure, trial registration, animal ethics, and AI/LLM disclosure); data availability agreement was 97.2%.

Pre-specified grouped contrast. For an exploratory test of whether change differed by marker type, the eight markers were grouped a priori into publication-infrastructure markers (data availability, funding disclosure, conflict-of-interest disclosure, AI/LLM disclosure) and study-conduct markers (ethics approval, informed consent, trial registration, animal ethics). The number of markers per article within each group was compared between 2021 and 2025 using Mann-Whitney U tests. Infrastructure markers increased (mean 1.58 to 2.23 per article; p<0.001) whereas study-conduct markers did not (1.14 to 1.32; p=0.098); among marker-article cells absent in 2021, 77 of 99 newly reported cells were infrastructure markers (Fisher exact p<0.001). This contrast is exploratory and ecological, performed on a convenience sample, and is reported in support of the descriptive completeness analysis rather than as a primary inferential result.

**Exploratory analyses**

An exploratory analysis assessed explicit mention of an institutional review board, ethics committee, or equivalent ethics oversight body, and a stricter ethics approval number or code marker. Article-level reporting completeness was calculated as the number of detected reporting markers per article; this descriptive count is not a validated quality score. A sensitivity version excluding animal ethics and AI/LLM disclosure (0-6 scale) was also calculated because these markers were rare and not applicable to many article types.

**Supplementary table and figure captions**

Supplementary Table S1. Prevalence of each reporting marker by publication year. Number and percentage of articles (of 120 per year) in which each of the eight markers was detected in 2021 and 2025, with 95% Wilson confidence intervals.

Supplementary Table S2. Number of articles with each reporting marker by journal and publication year (n=20 per journal-year). Cell values are counts of articles in which each marker was detected; animal ethics counts are after quality-control review.

Supplementary Figure S1. Distribution of reporting completeness by publication year. Percentage of articles with each number of detected reporting markers (0-8 scale) in 2021 and 2025, with dashed lines indicating annual means (2.73 and 3.55). This exploratory measure represents reporting density and should not be interpreted as a validated quality score.

Supplementary Figure S2. Sensitivity analysis of reporting completeness excluding animal ethics and AI/LLM disclosure (0-6 scale), with dashed lines indicating annual means (2.71 and 3.42). This sensitivity analysis was performed because these markers were rare and not applicable to many article types.

Supplementary Figure S3. Data availability statement reporting by publisher group, 2021 vs 2025. Percentage of articles with a detected data availability statement in each publisher group: Springer Nature (Clinical Oral Investigations), Wiley (International Journal of Paediatric Dentistry), Sage (Journal of Dental Research), and Elsevier (three journals combined). Significance markers indicate Fisher exact p-values.
